# The association of dietary patterns with incident chronic kidney disease and kidney function decline among middle-aged Korean adults: a cohort study

**DOI:** 10.4178/epih.e2023037

**Published:** 2023-03-21

**Authors:** Jialei Fu, Sangah Shin

**Affiliations:** Department of Food and Nutrition, Chung-Ang University, Anseong, Korea

**Keywords:** Diet healthy, Kidney diseases, Korea, Adults

## Abstract

**OBJECTIVES:**

This study assessed the association of dietary patterns with the incidence of chronic kidney disease (CKD) and kidney function decline among Korean adults.

**METHODS:**

Data were collected from the records of 20,147 men and 39,857 women who participated in the Health Examinees study. Principal component analysis was used to identify 3 dietary patterns (prudent, flour-based food and meat, and white rice-based), and CKD risk was defined using the Epidemiology Collaboration equation for estimated glomerular filtration rate: (eGFR) <60 mL/min/1.73 m^2^. A kidney function decline was defined as a >25% decrease in eGFR from baseline.

**RESULTS:**

During the 4.2-year follow-up, 978 participants developed CKD and 971 had a 25% decline in kidney function. After adjusting for potential impact variables, compared with the lowest quartile of the prudent dietary pattern, participants in the highest quartile had a 37% lower risk of kidney function decline among men (hazard ratio [HR], 0.63; 95% confidence interval [CI], 0.47 to 0.85); while higher adherence to the flour-based food and meat dietary pattern was associated with an increased risk of CKD in both men (HR, 1.63; 95% CI, 1.22 to 2.19) and women (HR, 1.47; 95% CI, 1.05 to 2.05) as well as a decline in kidney function in both men (HR, 1.49; 95% CI, 1.07 to 2.07) and women (HR, 1.77; 95% CI, 1.33 to 2.35).

**CONCLUSIONS:**

Although a higher adherence to the prudent dietary pattern was inversely associated with the risk of kidney function decline in men, there was no association with CKD risk. In addition, a higher adherence to the flour-based food and meat dietary pattern increased the risk of CKD and kidney function decline. Further clinical trials are needed to confirm these associations.

## GRAPHICAL ABSTRACT

[Fig f3-epih-45-e2023037]

## INTRODUCTION

Chronic kidney disease (CKD) is a global public health issue and a complex progressive disease. It has been predicted that CKD will become the fifth leading cause of death by 2040 [1]. Currently, CKD affects 850 million people, or 10% of the global population [2]. In Korea, the prevalence of CKD has been reported as 10.85%, and 2.03% of deaths were attributed to CKD in 2018 [3]. Globally, the leading causes of CKD are diabetes mellitus and hypertension [4]. Unmodifiable factors, such as environmental exposure, infection, and genetic risk, may also contribute to CKD and, as these risk factors increase, the incidence of CKD will also increase [4]. However, CKD is largely preventable and treatable, and diet is a key factor in the prevention and management of CKD [5].

The relationship of nutrition and dietary patterns to chronic diseases (cardiovascular diseases, diabetes, and hypertension) has been discussed in an increasing number of epidemiological studies. Nutrition and dietary patterns are modifiable risk factors that can be targeted to prevent or slow the progression of CKD [6]. Rather than focusing on individual nutrients, dietary patterns encompass the overall food group intake and may better reflect the potential synergy or antagonism between food groups than individual nutrient analyses [7]. The data from an observational survey indicated an increased risk of CKD among Chinese adults aged 49-59 who followed a Western dietary pattern, whereas a vegetable-grain dietary pattern was associated with reduced risk [8]. Additionally, the Chronic Renal Insufficiency Cohort (CRIC) study of American adults demonstrated that following a healthy dietary pattern can benefit kidney function [9]. However, the association between dietary patterns and the incidence of CKD, particularly kidney function decline, in Korean adults has not yet been explored. Therefore, to address this gap, we investigated the association of dietary patterns with CKD incidence and kidney function decline in Korean adults.

## MATERIALS AND METHODS

### Study population

The Health Examinees (HEXA) study was a large-scale community-based cohort study of Korean adults aged > 40 years. The baseline assessment was conducted from 2004 to 2013, and 173,357 participants were recruited. From 2012 to 2016, approximately 65,642 participants completed the second assessment. The objective design of the HEXA study has been described in previous reports [10,11]. For the present study, we excluded those participants without a record of biomarker values at baseline, follow-up data, or dietary data (2,711 participants). We further excluded those with CKD or a history of kidney-related disease at baseline (1,151 participants). Another 1,697 participants were excluded due to implausible energy intake (< 800 or ≥ 4,000 kcal/day for men; < 500 or ≥ 3,500 kcal/day for women) [12], and 81 participants without height or weight data were excluded. Our final sample included 60,004 participants (Supplementary Material 1).

### Dietary assessment

Dietary intake was assessed using a validated semiquantitative food frequency questionnaire (FFQ) containing 106 food items at baseline and follow-up. The participants were asked to recall the frequency of each food item consumed over the past 12 months and the portion size. Daily nutrient intake and energy intake were derived from the Korean food composition tables and were linked to consumption frequency and portion size for each food item [13].

### Identification of dietary patterns

The 106 food items were divided into 22 food groups, as detailed in Supplementary Material 2. Principal component analysis was used to identify the dietary patterns based on the total intake of the 22 food groups at baseline. For factor analysis, which is a data-driven approach, the Kaiser–Meyer–Olkin test was applied to assess the adequacy of sample sizes; acceptable values were found in this study (men: 0.77, women: 0.80). The varimax rotation was adopted for greater interpretability. Food groups with high factor loadings (absolute value ≥ 0.3) were regarded as the main contributors to each dietary pattern. As the scree plot shows (Supplementary Materials 3 and 4), 3 dietary patterns were extracted (determined by an eigenvalue > 1.5) and were labeled based on the characteristics of the major factor loadings: the prudent pattern, the flour-based food and meat pattern, and white rice pattern. Based on the scores, the dietary patterns were categorized into quartiles, with quartile 1 regarded as the reference. The extracted dietary patterns explained 32.55% and 32.67% of the dietary in- take among men and women, respectively (Supplementary Material 5).

### Determining outcomes

In this study, the primary outcome was CKD incidence, and the secondary outcome was kidney function decline. The estimated glomerular filtration rate (eGFR) was used to evaluate kidney function and was calculated using the CKD Epidemiology Collaboration equation [14] as follows:

If subjects are man,


$$\mathrm{eGFR}=141\times {\left(\frac{\mathrm{Serum}\;\mathrm{creatinine}\left[\mathrm{Scr}\right]}{0.9}\right)}^{\begin{array}{c} -0.411\left(\mathrm{If}\;\mathrm{Scr}\leq 0.9\;\mathrm{mg}/\mathrm{dL}\right)\\ -1.209\left(\mathrm{If}\;\mathrm{Scr}>0.9\mathrm{mg}/\mathrm{dL}\right) \end{array}}\times 0.993^{\mathrm{Age}}$$

If subjects are woman,


$$\mathrm{eGFR}=144\times {\left(\frac{\mathrm{Scr}}{0.7}\right)}^{\begin{array}{c} -0.329\left(\mathrm{IF}\;\mathrm{Scr}\leq 0.7\mathrm{mg}/\mathrm{dL}\right)\\ -1.209\left(\mathrm{IF}\;\mathrm{Scr}>0.7\mathrm{mg}/\mathrm{dL}\right) \end{array}}\times 0.993^{\mathrm{Age}}$$

Subjects with a follow-up eGFR below 60 mL/min/1.73 m^2^ were defined as CKD [15]. An eGFR decline from the baseline of > 25% was defined as kidney function decline. In this study, eGFR was only examined during the baseline and follow-up periods. Person-years were calculated from the baseline date to the date of the follow-up assessment or the date of examination in new-onset cases, whichever occurred first.

### Covariate assessment

Socio-demographic and health-related characteristics were assessed based on the answers to self-administered questionnaires. Data on age, body mass index (BMI), education level, physical activity, alcohol consumption, smoking status, total energy intake, serum creatinine and serum uric acid levels, and comorbidities were collected at baseline. Education level referred to the highest level of education attained and was classified as middle school, high school, or college. Physical activity was defined as “active” based on an affirmative answer to the following questions: “Do you exercise regularly to the point of sweating?” or “Do you exercise more than 5 days per week?” Smoking status was classified as non-smoker, former smoker, or current smoker. Alcohol consumption status was classified as non-drinker (including former drinker) or current drinker. Biomarkers (serum creatinine and serum uric acid levels) were obtained from a blood test. A comorbidity was considered present if the participant had one of the following diseases: diabetes, hypertension, and dyslipidemia.

### Statistical analysis

Continuous variables were expressed as mean± standard error or median (25 and 75%), and categorical variables were expressed as frequency (percentage). Cox proportional hazards models were used to identify associations between the 3 dietary patterns and the risk of CKD and kidney function decline. Hazard ratios (HRs) with 95% confidence intervals (CIs) were calculated across the quartiles of dietary pattern scores and were adjusted by 3 multivariable models. Model 1 was only adjusted for age. Model 2 was further adjusted for BMI, education level, physical activity, alcohol consumption, and smoking status. Model 3 was adjusted for all variables in model 2 as well as for diabetes, hypertension, dyslipidemia, proteinuria, and hyperuricemia.

Subgroup analyses were performed to explore potential confounders in the associations between the dietary patterns and the risk of CKD and kidney function decline. The likelihood ratio test was used to determine the interaction p-value. Among the potential confounders, socio-demographic characteristics (age, BMI), lifestyle behaviors (physical activity status, alcohol consumption, and smoking status [only observed in men]), and comorbidities were considered. All statistical analyses were performed using SAS version 9.4 (SAS Institute Inc., Cary, NC, USA). Figures were constructed using Stata MP version 17.0 (StataCorp., College Station, TX, USA). A two-tailed p-value < 0.05 was considered to indicate statistical significance.

### Ethics statement

This study was approved by the Ethics Committee of Korean Health and the institutional review board of each participating hospital (IRB No. E-1503-103-657), and was conducted in accordance with the guidelines of the Declaration of Helsinki. Written informed consent was obtained from all participants.

## RESULTS

### Dietary patterns

The factor loadings of the 3 dietary patterns by sex are presented in Supplementary Material 5. The distribution of factor loadings was found to be similar in men and women. The major contributors to the prudent dietary pattern were green and yellow vegetables, light-colored vegetables, mushrooms, fish and shellfish, seaweed, kimchi, soybean paste, potatoes, beans, tofu, soymilk, and fruits, and these contributors explained 17.02% and 17.45% of the variance in men and women, respectively. The main contributors to the flour-based food and meat dietary pattern were wheat flour and bread, white meat and its products, red meat and its products, noodles and dumplings, eggs, and beverages. The main contributors to the white rice dietary pattern were rice and other grains. The last 2 dietary patterns explained 6% to 8% of the variance in men and women (Supplementary Material 6).

### Characteristics of participants

The baseline characteristics of the 60,004 participants according to dietary pattern quartiles are shown in Table 1. In the highest quartile of the prudent dietary pattern, participants were older and more physically active, and the proportions of alcohol drinkers and smokers were lower. In contrast, participants in the highest quartile of the flour-based food and meat dietary pattern and the white rice dietary pattern tended to be younger and less physically active, and the proportions of alcohol drinkers and smokers were higher. Moreover, participants with a high adherence to the prudent dietary pattern or the flour-based food and meat dietary pattern had a higher level of education and higher energy intake, while participants with higher adherence to the white rice dietary pattern were more likely to have a low education level and lower energy intake.

### Association of dietary patterns with kidney outcomes

Over a median follow-up of 4.2 years, there were 978 new cases of CKD and 971 with a 25% kidney function decline. After multivariable adjustment, our results indicated that higher adherence to the prudent dietary pattern could lower the risk of kidney function decline in men by 37% (HR, 0.63; 95% CI, 0.47 to 0.85) when compared with the lowest quartiles, whereas there was no significant association between the prudent dietary pattern and CKD risk (p> 0.05; Table 2). The associations of the flour-based food and meat dietary pattern with the risk of CKD and kidney function decline are presented in Table 3. Men in the highest quartile of the flour-based food and meat dietary pattern had a 63% higher risk (HR, 1.63; 95% CI, 1.22 to 2.19) of CKD than those in the lowest quartile, while women had a 47% higher risk (HR, 1.47; 95% CI, 1.05 to 2.05). Similar results were obtained for the risk of kidney function decline (men: HR, 1.49; 95% CI, 1.07 to 2.07; and women: HR, 1.77; 95% CI, 1.33 to 2.35). No statistically significant associations were observed between the white rice dietary pattern and CKD risk (men: HR, 1.05; 95% CI, 0.81 to 1.36; and women: HR, 0.97; 95% CI, 0.71 to 1.33) or the risk of kidney function decline (men: HR, 1.04; 95% CI, 0.77 to 1.40; and women: HR, 1.07; 95% CI, 0.82 to 1.40) (Supplementary Material 7).

### Subgroup analysis

**Figure 1.**
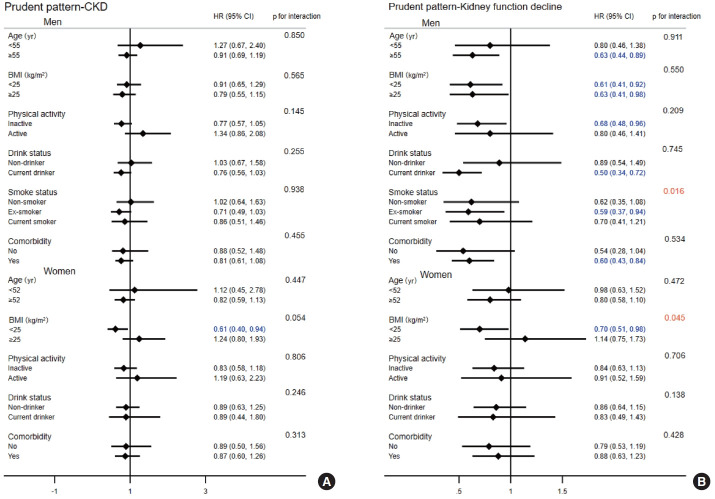
Stratified analyses of the associations between the prudent dietary pattern and the risk of CKD (A) and kidney function decline (B) by gender. Adjusted for age at baseline, BMI, energy intake, education level, physical activity level (yes or no), alcohol consumption, smoking status, and comorbidities; stratified variables were not adjusted. CKD, chronic kidney disease; HR, hazard ratio; CI, confidence interval; BMI, body mass index.

**Figure 2.**
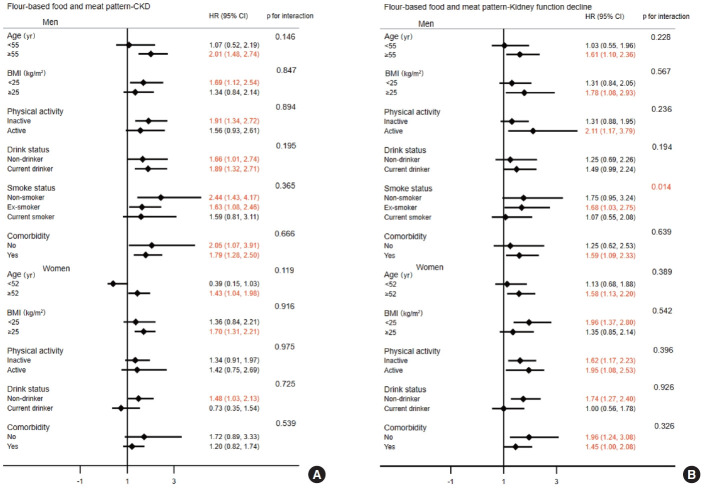
Stratified analyses of the associations between the flour-based food and meat dietary pattern and the risk of CKD (A) and kidney function decline (B) by gender. Adjusted for age at baseline, BMI, energy intake, education level, physical activity level (yes or no), alcohol consumption, smoking status, and comorbidities; stratified variables were not adjusted. CKD, chronic kidney disease; HR, hazard ratio; CI, confidence interval; BMI, body mass index.

The interactions among age, BMI, physical activity, smoking status, drinking status, comorbidity, and the prudent dietary pattern in relation to the risk of CKD and kidney function decline are presented in Figure 1. The results indicated significant interaction effects between smoking status in men (p for interaction= 0.016) and BMI in women (p for interaction= 0.045) and the prudent dietary pattern in relation to the risk of kidney function decline, whereas no interaction effect was observed for the risk of CKD. Participants whose BMI was < 25 kg/m^2^ had a 39% and 30% reduced risk of CKD (HR, 0.61; 95% CI, 0.40 to 0.94) and kidney function decline (HR, 0.70; 95% CI, 0.51 to 0.98) among women, respectively. For the flour-based food and meat dietary pattern, there was a significant interaction effect between smoking status and kidney function decline in men (p for interaction= 0.014; Figure 2). Although the white rice dietary pattern showed no significant associations with the risk of CKD or kidney function decline, we still conducted a subgroup analysis (Supplementary Material 8). However, no interactions were found (all p for interaction > 0.05).

## DISCUSSION

In this cohort of 20,147 men and 39,857 women, we observed that the prudent dietary pattern was inversely associated with a risk of kidney function decline among Korean men, and there was no association among women. However, a significant factor interaction was found between the prudent dietary pattern and BMI for the risk of kidney function decline among women. Participants whose BMI was < 25 kg/m^2^ in the highest quartiles had a 39% and 30% lower risk of CKD and kidney function decline, respectively, compared with those in the lowest quartiles. In contrast, the flour-based food and meat dietary pattern was positively associated with the risk of CKD and kidney function decline. To our knowledge, this is the first cohort study to investigate the association of dietary patterns with CKD risk among Korean adults. We also considered the effect of dietary patterns on the risk of kidney function decline as a secondary analysis.

The prudent dietary pattern was characterized by vegetables, fruits, fish, and soy foods and shared several characteristics with other healthy diet patterns, such as the Dietary Approaches to Stop Hypertension (DASH) diet, and the Healthy Eating Index [9,16]. Consistent with our findings, previous studies have reported the benefit of the prudent dietary pattern in reducing the risk of kidney function decline [17,18]. A United States-based prospective cohort study found that the DASH dietary pattern appeared to lower the risk of kidney function decline by > 40% [18] due to its high proportion of fruits and vegetables. Growing evidence indicates the benefits of fiber, potassium, and bicarbonate in fruits and vegetables in preventing the risk of kidney function decline [19-21].

In contrast, our findings showed that this dietary pattern (prudent; high in fruits and vegetables) was not associated with CKD in either men or women [22]. In 2018, an Iran-based study, with 6.1 years of follow-up, reported that a lacto-vegetarian dietary pattern was associated with a 43% decrease in incident CKD [22]. Shi et al. [23] showed that a modern dietary pattern could reduce the risk of CKD by 50%. In the present study, we assessed the effect of diabetes and hypertension (2 major causes of CKD) [4] on the prudent dietary pattern and CKD, but the results did not change when we adjusted for these complications. We surmise that this might be due to the high sodium intake in this dietary pattern. The prudent dietary pattern in Korea is unique compared to other countries because the vegetable intake includes fermented foods, such as kimchi, which require abundant salting during the fermentation process. Several studies have shown that salt intake increases the risk of CKD [24-26]. A cohort study from the same database as our study indicated that non-fermented vegetable consumption was directly associated with a 20% reduced risk of CKD, whereas no association was found for fermented vegetable consumption [27]. Therefore, we speculate that there was no association between this dietary pattern and the risk of CKD due to the synergy between food groups. Notably, after we stratified participants according to BMI levels, we found that greater adherence to the prudent dietary pattern was beneficial, with a lower risk of CKD and kidney function decline in participants whose BMI was < 25 kg/m^2^. There is strong evidence for the direct causal effect of BMI on the risk of kidney function decline and CKD [28,29]. Additionally, participants with a low BMI were better at following a healthy diet than those with a high BMI [30]. Therefore, compared with participants whose BMI was ≥ 25 kg/m^2^, participants with lower BMIs had a reduced risk of CKD and kidney function decline.

The flour-based food and meat dietary pattern was characterized by meat products, noodles, and beverages, a diet commonly regarded as unhealthy. Our findings support previous studies that showed that this dietary pattern can increase the risk of CKD and kidney function decline [8,18,22]. In 2021, He et al. [31] conducted a meta-analysis of 17 studies and found that the Western dietary pattern was linked to an increased risk of CKD. Another review described animal fat, protein, sugar, and sodium chloride as the 4 major risk factors of the Western dietary pattern that contribute to kidney function decline and CKD [32]. There are a few explanations for this association. First, the high intake of animal-derived foods associated with this dietary pattern is linked to microbial alterations and the production of inflammatory metabolites in vivo [33]. Evidence has shown that a Western diet may cause systemic inflammation, a condition intrinsically linked to CKD and detrimental to kidney function [34-36]. Second, many reviews and epidemiological studies have emphasized the high protein intake in a diet rich in red, white, and processed meat. Protein metabolism can increase the burden on kidneys, ultimately contributing to the development of kidney-related diseases [37,38]. Third, in this study, physical activity was lower in those with greater adherence to this dietary pattern. A longitudinal study in Taiwan selected 199,421 participants and reported that high physical activity decreased the risk of CKD [39]. Some studies have demonstrated that low levels of physical activity lead to insulin resistance, obesity, a reduction in adipocytokines, and an increase in visceral adipocytes that damage the renal vascular system and increase angiotensinogen synthesis, subsequently adversely affecting the kidneys [40-42]. Fourth, high fructose intake has been positively correlated with an increased risk of CKD [43,44]. A recent experiment in mice also demonstrated that fructose can induce the development of CKD because high fructose consumption led to the induction of the carbohydrate-response element-binding protein beta transcription factor and overexpression of its downstream genes [45]. Therefore, as the US National Institute of Diabetes and Digestive and Kidney Disease stated, to maintain kidney health, we should make healthy food choices [46] such as fresh fruits and vegetables, and cut back on added sugars and salty foods.

The main contributors to the white rice dietary pattern were rice and other grains. Our study found no statistical significance between this dietary pattern and the risk of CKD or kidney function decline. Our results were similar to those of studies conducted in China and Iran on 2 dietary patterns high in grains, the traditional southern Chinese dietary pattern and the traditional Iranian dietary pattern [8,22]. The percentages of refined and whole grains possibly influenced these associations. Whole grains have been shown to have a beneficial effect on renal function and CKD, while a negative correlation has been shown between refined grains and eGFR [47]. However, the white rice-based dietary pattern in our study did not distinguish the ratios of whole grains to refined grains. Taken together, the functional effect of these 2 types of grains on CKD and renal function led to the loss of correlation between this dietary pattern and CKD.

There were some potential limitations in this study. First, the follow-up rate in this study was relatively low. Second, the FFQ in our study was self-administrated, leading to inevitable report bias and recall bias. However, the dietary intake data was also validated by 24-hour recall to minimize errors as much as possible. Third, we only considered food consumption at baseline in our dietary intake assessment, which did not account for changes in dietary intake over the follow-up period. However, because the follow-up period was short (4.2 years), changes in dietary intake were less likely. Fourth, our team subjectively determined the food groupings, possibly leading to selection bias. A strength of our study was that a large sample was analyzed, increasing the reliability of the results. Furthermore, to our knowledge, this is the first study to assess the association between dietary patterns and the risk of CKD and kidney function decline in Korean adults.

In conclusion, the prudent dietary pattern was associated with a lower risk of kidney function decline in Korean men, whereas the flour-based food and meat dietary pattern was associated with an increased risk of CKD and kidney function decline in all participants. Our findings provide reliable evidence of the effect of dietary patterns on kidney function outcomes, with a focus on Korean adults, and provide an attractive therapeutic target for the prevention of CKD progression and kidney function decline. More clinical trials are warranted to confirm these associations.

## DATA AVAILABILITY

Data described in the manuscript, code book, and analytic code will be made publicly and freely available without restriction.

## Figures and Tables

**Figure f3-epih-45-e2023037:**
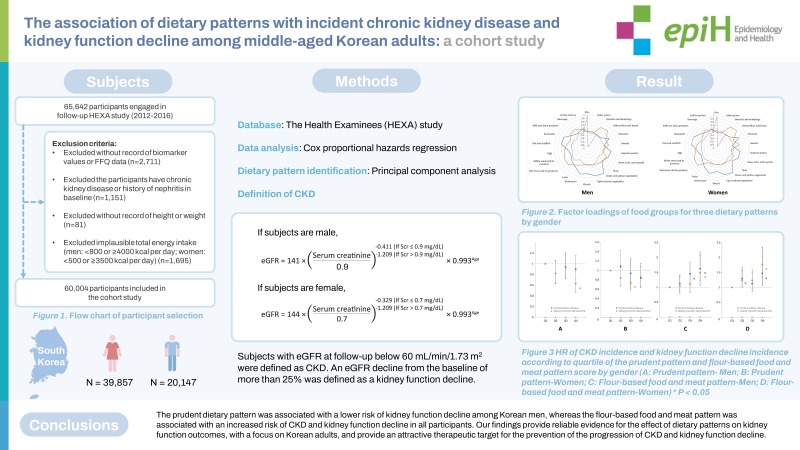


**Table 1.** Baseline participant characteristics according to the 3 dietary pattern quartiles and by gender

Characteristics			Quartile of dietary pattern score									
			Men					Women				
			Q1 (n=5,036)	Q2 (n=5,037)	Q3 (n=5,037)	Q4 (n=5,037)	p for trend	Q1 (n=9,964)	Q2 (n=9,964)	Q3 (n=9,965)	Q4 (n=9,964)	p for trend
Prudent dietary pattern												
	Age (yr)		53.85±0.12	54.85±0.12	55.37±0.12	55.75±0.12	<0.001	52.25±0.08	52.92±0.08	53.01±0.08	52.99±0.07	<0.001
	BMI (kg/m^2^)		24.29±0.04	24.36±0.04	24.48±0.04	24.58±0.04	<0.001	23.48±0.03	23.59±0.03	23.61±0.03	23.69±0.03	<0.001
	Education level						0.013					<0.001
		≤Middle school	1,061 (21.2)	1,115 (22.4)	1,073 (21.5)	963 (19.3)		3,823 (38.8)	3,804 (38.5)	3,524 (35.7)	3,061 (31.1)	
		High school	2,029 (40.6)	2,014 (40.45)	2,041 (40.9)	2,053 (41.2)		4,141 (42.0)	4,188 (42.4)	4,372 (44.3)	4,721 (48.0)	
		≥College	1,903 (38.1)	1,850 (37.16)	1,870 (37.5)	1,965 (39.4)		1,888 (19.2)	1,878 (19.0)	1,961 (19.9)	2,053 (20.9)	
	Physical activity (yes)		938 (18.7)	1,122 (22.32)	1,214 (24.2)	1,476 (29.4)	<0.001	1,644 (16.5)	1,916 (19.3)	2,209 (22.2)	2,563 (25.8)	<0.001
	Alcohol consumption (yes)		3,553 (70.7)	3,659 (72.83)	3,662 (73.1)	3,658 (72.8)	0.021	3,068 (30.9)	2,944 (29.6)	2,910 (29.3)	2,815 (28.4)	0.002
	Smoking status (yes)		1,419 (28.2)	1,401 (27.91)	1,325 (26.4)	1,276 (25.4)	0.007	217 (2.2)	160 (1.6)	140 (1.4)	137 (1.4)	<0.001
	Total energy (kcal/day)		1,636.41±6.02	1,734.51±5.56	1,861.79±5.84	2,099.13±6.86	<0.001	1,425.39±4.15	1,567.33±3.99	1721.35±4.15	2,007.07±5.05	<0.001
	Serum creatinine (mg/dL)		0.96±0.00	0.96±0.00	0.96±0.00	0.95±0.00	0.003	0.72±0.00	0.72±0.00	0.73±0.00	0.73±0.00	0.167
	eGFR (mL/min/1.73 m^2^)		89.22±0.17	88.70±0.17	88.42±0.17	88.44±0.17	0.053	94.26±0.13	93.62±0.13	93.44±0.13	93.35±0.12	<0.001
	Serum uric acid (mg/dL)		5.62±0.02	5.62±0.02	5.59±0.02	5.63±0.02	0.956	4.13±0.01	4.15±0.01	4.15±0.01	4.18±0.01	<0.001
Flour-based food and meat dietary pattern												
	Age (yr)		57.98±0.11	55.56±0.11	54.00±0.12	52.28±0.12	<0.001	55.77±0.07	53.48±0.07	51.96±0.08	49.96±0.07	<0.001
	BMI (kg/m^2^)		24.20±0.04	24.32±0.04	24.52±0.04	24.66±0.04	<0.001	23.80±0.03	23.59±0.03	23.52±0.03	23.46±0.03	0.003
	Education level						<0.001					<0.001
		≤Middle school	1,473 (29.6)	1,148 (23.04)	902 (18.1)	689 (13.8)		5,219 (53.0)	3,832 (38.9)	3,020 (30.7)	2,141 (21.7)	
		High school	2,034 (40.9)	2,053 (41.21)	2,039 (40.8)	2,011 (40.3)		3,543 (35.9)	4,341 (44.1)	4,701 (47.7)	4,837 (49.0)	
		≥College	1,464 (29.4)	1,781 (35.75)	2,054 (41.1)	2,289 (45.9)		1,093 (11.1)	1,670 (17.0)	2,127 (21.6)	2,890 (29.3)	
	Physical activity (yes)		1,248 (24.8)	1,215 (24.24)	1,153 (22.9)	1,134 (22.6)	0.025	2,289 (23.0)	2,055 (20.7)	2,070 (20.8)	1,918 (19.3)	<0.001
	Alcohol consumption (yes)		3,425 (68.1)	3,616 (72.10)	3,731 (74.3)	3,760 (74.9)	<0.001	1,877 (18.9)	2,768 (27.9)	3,315 (33.4)	3,777 (38.0)	<0.001
	Smoking status (yes)		1,076 (21.4)	1,300 (25.93)	1,445 (28.8)	1,600 (31.9)	<0.001	101 (1.0)	144 (1.4)	187 (1.9)	222 (2.2)	<0.001
	Total energy (kcal/day)		1,523.23±4.59	1,675.6±4.61	1,870.84±5.09	2,262.17±6.56	<0.001	1,420.71±3.89	1,523.15±3.80	1,695.75±3.94	2,081.55±4.90	<0.001
	Serum creatinine (mg/dL)		0.95±0.00	0.96±0.00	0.96±0.00	0.96±0.00	0.003	0.72±0.00	0.73±0.00	0.73±0.00	0.73±0.00	0.228
	eGFR (mL/min/1.73 m^2^)		87.28±0.17	88.37±0.17	89.13±0.18	89.99±0.18	<0.001	91.90±0.12	93.12±0.12	94.18±0.13	95.48±0.13	<0.001
	Serum uric acid (mg/dL)		5.53±0.02	5.58±0.02	5.65±0.02	5.70±0.02	<0.001	4.17±0.01	4.15±0.01	4.14±0.01	4.15±0.01	0.143
White rice dietary pattern												
	Age (yr)		55.11±0.12	55.65±0.11	55.71±0.12	53.35±0.12	<0.001	52.14±0.08	54.25±0.08	52.52±0.07	52.26±0.08	0.001
	BMI (kg/m^2^)		24.45±0.04	24.38±0.04	24.52±0.04	24.35±0.04	0.459	23.48±0.03	23.87±0.03	23.44±0.03	23.59±0.03	0.163
	Education level						<0.001					<0.001
		≤Middle school	866 (17.3)	1,106 (22.23)	1,140 (22.9)	1,100 (22.1)		2,982 (30.3)	4,563 (46.3)	2,985 (30.3)	3,682 (37.4)	
		High school	1,972 (39.5)	2,071 (41.62)	2,058 (41.3)	2,036 (40.8)		4,606 (46.7)	3,907 (39.6)	4,662 (47.3)	4,247 (43.1)	
		≥College	2,153 (43.1)	1,799 (36.15)	1,788 (35.9)	1,848 (37.1)		2,265 (23.0)	1,384 (14.0)	2,217 (22.5)	1,914 (19.4)	
	Physical activity (yes)		1,461 (29.1)	1,211 (24.10)	1,196 (23.8)	882 (17.6)	<0.001	2,220 (22.3)	2,105 (21.2)	2,238 (22.5)	1,769 (17.8)	<0.001
	Alcohol consumption (yes)		3,495 (69.7)	3,635 (72.31)	3,707 (73.7)	3,695 (73.7)	<0.001	2,895 (29.2)	2,570 (25.9)	3,136 (31.6)	3,136 (31.7)	<0.001
	Smoking status (yes)		1,100 (21.9)	1,194 (23.76)	1,466 (29.1)	1,661 (33.2)	<0.001	96 (1.0)	114 (1.1)	196 (2.0)	248 (2.5)	<0.001
	Total energy (kcal/day)		2,147.88±6.15	1,775.4±4.80	1,668.68±6.25	1,739.99±6.69	<0.001	2004.87±4.19	1,761.63±3.48	1,499.59±4.47	1,455.08±5.03	<0.001
	Serum creatinine (mg/dL)		0.96±0.00	0.96±0.00	0.96±0.00	0.95±0.00	0.538	0.73±0.00	0.72±0.00	0.73±0.00	0.72±0.00	0.697
	eGFR (mL/min/1.73 m^2^)		88.48±0.17	88.15±0.17	88.25±0.17	89.89±0.18	0.029	94.09±0.13	92.80±0.12	93.48±0.13	94.30±0.13	0.133
	Serum uric acid (mg/dL)		5.61±0.02	5.58±0.02	5.63±0.02	5.64±0.02	0.029	4.13±0.01	4.17±0.01	4.17±0.01	4.14±0.01	0.538

Values are presented as mean±standard error or number (%). Q1, quartile 1; Q2, quartile 2; Q3, quartile 3; Q4, quartile 4; BMI, body mass intake, eGFR, glomerular filtration rate.

**Table 2.** The hazard ratios of incident CKD and kidney function decline according to quartile of the prudent dietary pattern

Variables			Q1	Q2	Q3	Q4	p for trend
Men (n)			5,036	5,037	5,037	5,037	
	Person-years, total		24,716.9	24,181.7	24,133.4	24,424.1	
	Risk of CKD						
		Cases (n)	143	125	141	143	
		Crude	1.00 (reference)	0.94 (0.74, 1.20)	1.07 (0.85, 1.35)	1.04 (0.82, 1.31)	0.568
		Model 1	1.00 (reference)	0.85 (0.67, 1.08)	0.92 (0.73, 1.16)	0.87 (0.69, 1.10)	0.405
		Model 2	1.00 (reference)	0.85 (0.67, 1.09)	0.92 (0.73, 1.17)	0.88 (0.69, 1.13)	0.474
		Model 3	1.00 (reference)	0.82 (0.64, 1.05)	0.94 (0.74, 1.19)	0.90 (0.70, 1.15)	0.669
	Risk of kidney function decline ≥25%						
		Cases (n)	122	103	117	84	
		Crude	1.00 (reference)	0.91 (0.70, 1.19)	1.04 (0.81, 1.34)	0.72 (0.54, 0.95)	0.033
		Model 1	1.00 (reference)	0.87 (0.67, 1.13)	0.96 (0.75, 1.24)	0.65 (0.49, 0.86)	0.005
		Model 2	1.00 (reference)	0.87 (0.67, 1.13)	0.96 (0.74, 1.24)	0.65 (0.48, 0.88)	0.008
		Model 3	1.00 (reference)	0.82 (0.63, 1.07)	0.93 (0.72, 1.21)	0.63 (0.47, 0.85)	0.006
Women (n)			9,964	9,964	9,965	9,964	
	Person-years, total		48,159.3	48,308.9	48,615.5	50,828.7	
	Risk of CKD						
		Cases (n)	104	122	99	101	
		Crude	1.00 (reference)	1.19 (0.91, 1.54)	0.95 (0.72, 1.25)	0.84 (0.64, 1.11)	0.063
		Model 1	1.00 (reference)	1.10 (0.84, 1.43)	0.89 (0.68, 1.18)	0.85 (0.64, 1.11)	0.095
		Model 2	1.00 (reference)	1.10 (0.85, 1.43)	0.91 (0.69, 1.21)	0.86 (0.63, 1.17)	0.182
		Model 3	1.00 (reference)	1.09 (0.84, 1.42)	0.94 (0.71, 1.25)	0.85 (0.63, 1.15)	0.166
	Risk of kidney function decline ≥25%						
		Cases (n)	158	134	114	139	
		Crude	1.00 (reference)	0.86 (0.68, 1.08)	0.72 (0.56, 0.91)	0.76 (0.60, 0.95)	0.014
		Model 1	1.00 (reference)	0.82 (0.65, 1.03)	0.69 (0.54, 0.88)	0.74 (0.59, 0.93)	0.012
		Model 2	1.00 (reference)	0.85 (0.67, 1.07)	0.74 (0.58, 0.95)	0.85 (0.66, 1.10)	0.233
		Model 3	1.00 (reference)	0.84 (0.67, 1.06)	0.74 (0.58, 0.95)	0.83 (0.65, 1.08)	0.189

Values are presented as hazard ratio (95% confidence interval). Model 1: Adjusted for age; Model 2: Further adjusted for body mass index, energy intake, education level, physical activity level (yes or no), alcohol consumption status, and smoking status; Model 3: Further adjusted for diabetes, hypertension, dyslipidemia, proteinuria, and hyperuricemia. CKD, chronic kidney disease; Q1, quartile 1; Q2, quartile 2; Q3, quartile 3; Q4, quartile 4.

**Table 3.** The hazard ratios of incident CKD and kidney function decline according to quartile of the flour-based food and meat dietary pattern

Variables			Q1	Q2	Q3	Q4	p for trend
Men (n)			5,036	5,037	5,037	5,037	
	Person-years, total		25,093.6	24,353.4	24,114.6	23,894.5	
	Risk of CKD						
		Cases (n)	177	128	131	116	
		Crude	1.00 (reference)	0.79 (0.63, 1.00)	0.83 (0.66, 1.04)	0.76 (0.60, 0.96)	0.037
		Model 1	1.00 (reference)	1.04 (0.83, 1.31)	1.23 (0.98, 1.54)	1.34 (1.05, 1.70)	0.008
		Model 2	1.00 (reference)	1.10 (0.87, 1.39)	1.41 (1.11, 1.80)	1.76 (1.31, 2.36)	<0.001
		Model 3	1.00 (reference)	1.12 (0.89, 1.42)	1.45 (1.13, 1.86)	1.63 (1.22, 2.19)	<0.001
	Risk of kidney function decline ≥25%						
		Cases (n)	138	99	90	99	
		Crude	1.00 (reference)	0.79 (0.61, 1.02)	0.73 (0.56, 0.96)	0.83 (0.64, 1.08)	0.181
		Model 1	1.00 (reference)	0.92 (0.71, 1.19)	0.92 (0.70, 1.20)	1.15 (0.89, 1.50)	0.290
		Model 2	1.00 (reference)	0.98 (0.75, 1.27)	1.04 (0.78, 1.39)	1.48 (1.06, 2.05)	0.023
		Model 3	1.00 (reference)	1.04 (0.79, 1.35)	1.11 (0.83, 1.48)	1.49 (1.07, 2.07)	0.020
Women (n)			9,964	9,964	9,965	9,964	
	Person-years, total		50,243.3	48,895.5	48,181.4	48,592.2	
	Risk of CKD						
		Cases (n)	151	115	84	76	
		Crude	1.00 (reference)	0.82 (0.64, 1.05)	0.62 (0.48, 0.81)	0.53 (0.41, 0.70)	<0.001
		Model 1	1.00 (reference)	1.14 (0.89, 1.46)	1.01 (0.77, 1.33)	1.18 (0.89, 1.56)	0.370
		Model 2	1.00 (reference)	1.17 (0.92, 1.50)	1.05 (0.79, 1.39)	1.28 (0.92, 1.78)	0.207
		Model 3	1.00 (reference)	1.30 (1.02, 1.67)	1.13 (0.85, 1.49)	1.47 (1.05, 2.05)	0.048
	Risk of kidney function decline ≥25%						
		Cases (n)	167	140	113	125	
		Crude	1.00 (reference)	0.90 (0.72, 1.13)	0.76 (0.60, 0.96)	0.79 (0.62, 0.99)	0.029
		Model 1	1.00 (reference)	1.09 (0.87, 1.36)	1.01 (0.79, 1.28)	1.21 (0.95, 1.54)	0.162
		Model 2	1.00 (reference)	1.16 (0.92, 1.46)	1.16 (0.90, 1.49)	1.63 (1.23, 2.15)	0.001
		Model 3	1.00 (reference)	1.24 (0.99, 1.56)	1.24 (0.96, 1.59)	1.77 (1.33, 2.35)	<0.001

Values are presented as hazard ratio (95% confidence interval). Model 1: Adjusted for age; Model 2: Further adjusted for body mass index, energy intake, education level, physical activity level (yes or no), alcohol consumption status, and smoking status; Model 3: Further adjusted for diabetes, hypertension, dyslipidemia, proteinuria, and hyperuricemia. CKD, chronic kidney disease; Q1, quartile 1; Q2, quartile 2; Q3, quartile 3; Q4, quartile 4.

## SUPPLEMENTARY MATERIALS

Supplementary Material 1. Flow chart for participant selection

Supplementary Material 2. Food grouping used in the dietary pattern analyses

Supplementary Material 3. Scree plot and variance explained in factor analysis of men

Supplementary Material 4. Scree plot and variance explained in factor analysis of women

Supplementary Material 5. Factor loadings of food groups for three dietary patterns by gender

Supplementary Material 6. Food groups intakes (g/day) according to quartile of three dietary pattern scores found in Korean adults

Supplementary Material 7. Hazard ratio of CKD incidence and kidney function decline incidence according to quartile of the white rice pattern score

Supplementary Material 8. Stratified analyses of associations between three dietary patterns and CKD risk and kidney function decline risk by gender
