# Supplementary figures and images for: The association of dietary patterns with incident chronic kidney disease and kidney function decline among middle-aged Korean adults: a cohort study
Source: Epidemiol Health. 2023 Mar 21;45:e2023037. doi: 10.4178/epih.e2023037 (PMC10586924; doi:10.4178/epih.e2023037)

1    Supplementary Material 1 Flow chart for participant selection

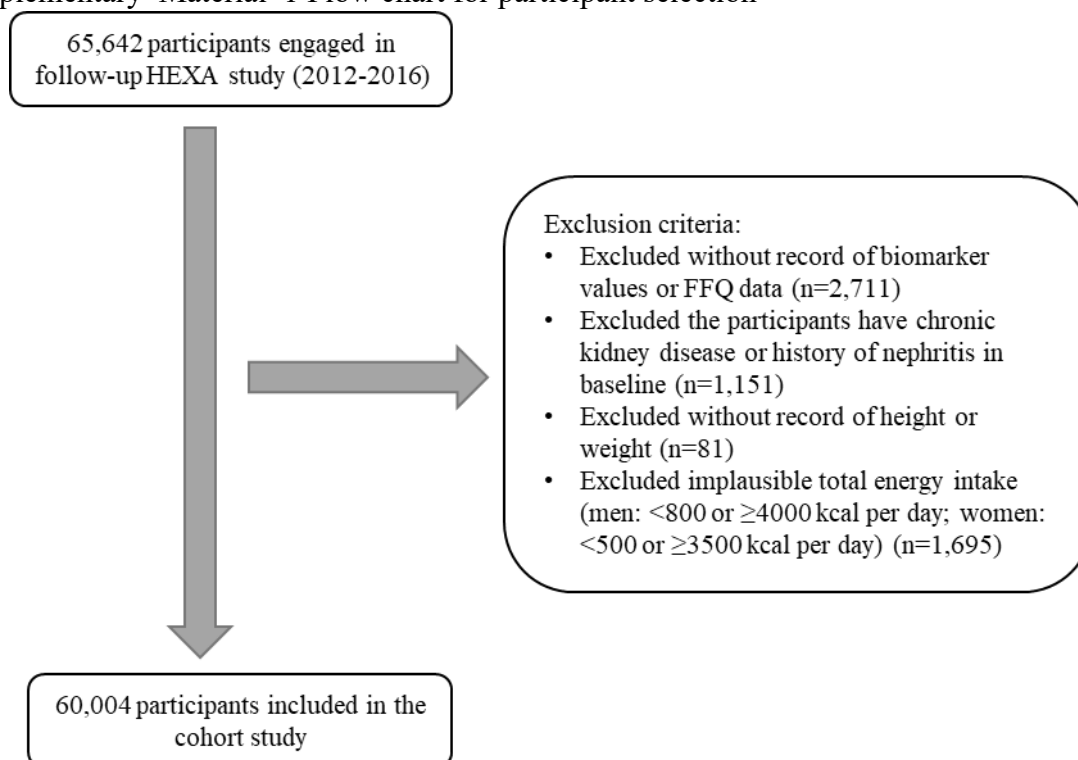

2  
3

Supplement: Supplementary Material 1. — Flow chart for participant selection [file epih-45-e2023037-Supplementary-1.pdf]

6    Supplementary   Material 3 Scree plot and variance explained in factor analysis of men

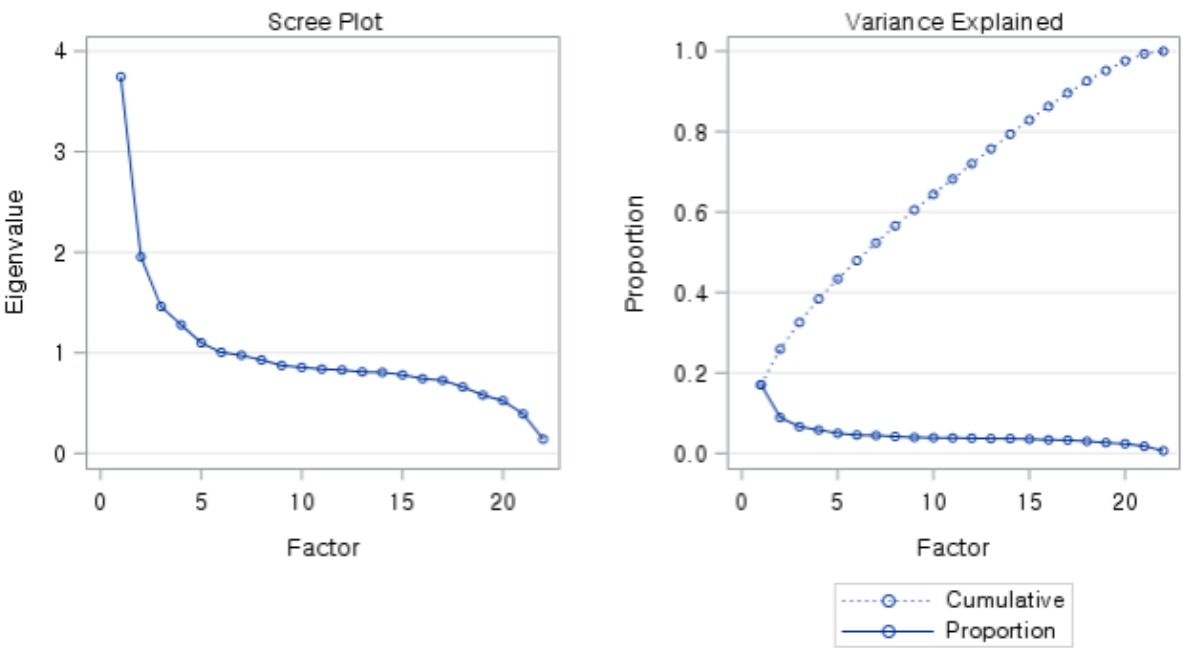

Supplement: Supplementary Material 3. — Scree plot and variance explained in factor analysis of men [file epih-45-e2023037-Supplementary-3.pdf]

8    Supplementary Material 4 Scree plot and variance explained in factor analysis of women

---

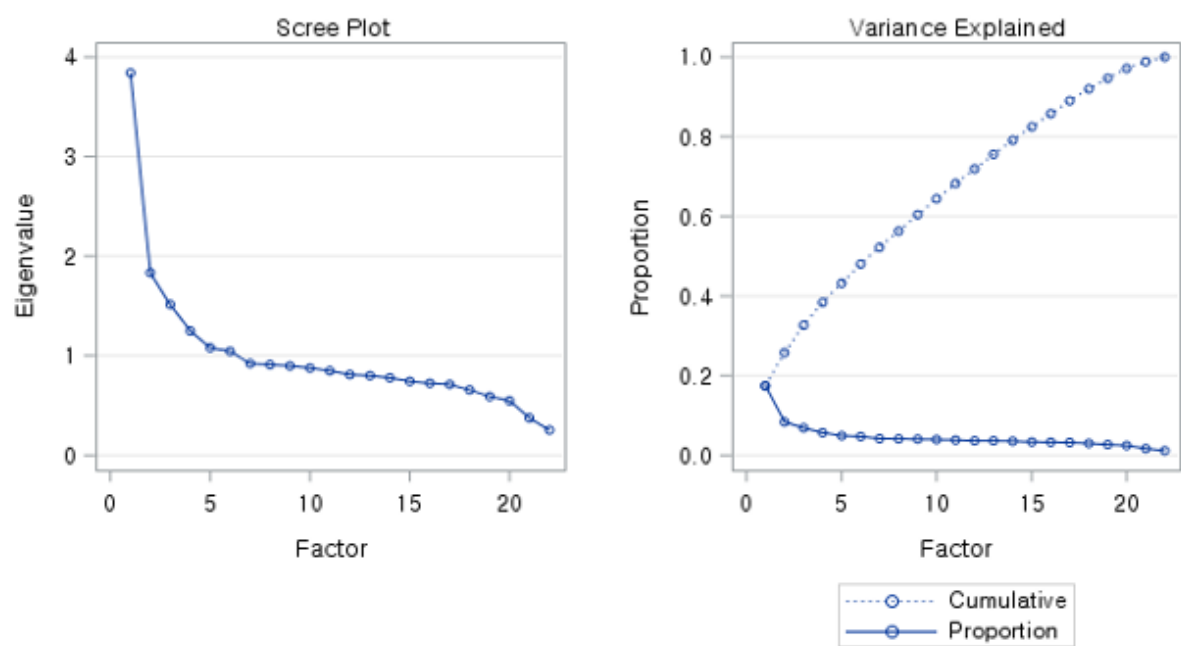

9

10

11

Supplement: Supplementary Material 4. — Scree plot and variance explained in factor analysis of women [file epih-45-e2023037-Supplementary-4.pdf]
