# Supplementary material for: The association of dietary patterns with incident chronic kidney disease and kidney function decline among middle-aged Korean adults: a cohort study
Source: Epidemiol Health. 2023 Mar 21;45:e2023037. doi: 10.4178/epih.e2023037 (PMC10586924; doi:10.4178/epih.e2023037)
Supplement: Supplementary Material 2. — Food grouping used in the dietary pattern analyses [file epih-45-e2023037-Supplementary-2.pdf]

Supplementary Material 2 Food grouping used in the dietary pattern analyses

**Food groups**	**Food items**
Rice	Cooked rice, rice cake, rice cake with soup, other rice cakes
Other grains	Cooked rice with soybean, cooked rice with other cereals, half and half cooked well-milled rice and rice with soybean, half and half cooked well-milled rice and rice with other cereals, parched cereal powder
Noodles and dumplings	Ramen, wheat noodles with soup, chajang myon, jambbong, buckwheat vermicelli, buckwheat noodle, dumpling, dumpling with soup
Wheat flour and bread	Cereals, corn flakes, loaf bread, sandwich, toast, bread with small red bean, other breads, cakes, choco pie, pizza, hamburger, cookie, cracker, snack
Potatoes	Starch jelly, potatoes, sweet potatoes, starch vermicelli
Sweets	Candy, chocolate, coffee sugar
Soybean pastes	Soup and stew with soybean paste, soybean paste
Bean, tofu, and soymilk	Legumes, tofu, soybean milk
Nuts	Nuts
Green and yellow vegetables	Spinach, lettuce, perilla leaf, vegetable wrap, vegetable salad, other green vegetables, pepper leaves, chamnamul, aster scaber, crown daisy, leek, water dropwort, cucumber, carrot, carrot juice, green pepper, pumpkin (immature), pumpkin (mature), pumpkin juice
Light-colored vegetables	Radish, salted radish, Korean cabbages, Korean cabbage soup, deoduck, doraji, bean sprouts, bracken, sweet potato stalk, stem of taro
Kimchi	Kimchi, Korean cabbage, kakdugi, small-radish kimchi, kimchi with water, radish with water, other kimchi, Korean-style pickles
Mushroom	Oyster mushroom, other mushrooms
Fruits	Strawberry, muskmelon, melon, watermelon, peach, plum, banana, persimmon, persimmon, tangerine, pear, pear juice, apple, apple juice, orange, orange juice, grape, grape juice, tomato, cherry tomato, tomato juice
Red meat and its products	Pork, ham, sausage, edible viscera, steak, beef, dog meat, beef soup, beef soup with vegetables
White meat and its products	Chicken
Eggs	Eggs
Fish and shellfish	Sashimi, mackerel, Pacific saury, Spanish mackerel, hairtail, eel, yellow croaker, sea bream, flatfish, Alaska pollock, cuttlefish, octopus, dried anchovies, tuna, salt-fermented fish, clam, whelk, oyster, crab, shrimp, fish paste, crab-flavored
Seaweeds	Laver, kelp, sea mustard
Milk and dairy products	Milk, yogurt, ice cream, cheese, coffee cream
Beverage	Carbonated drinks, other drinks
Coffee and tea	Coffee, green tea
