# Supplementary material for: The association of dietary patterns with incident chronic kidney disease and kidney function decline among middle-aged Korean adults: a cohort study
Source: Epidemiol Health. 2023 Mar 21;45:e2023037. doi: 10.4178/epih.e2023037 (PMC10586924; doi:10.4178/epih.e2023037)
Supplement: Supplementary Material 5. — Factor loadings of food groups for three dietary patterns by gender [file epih-45-e2023037-Supplementary-5.pdf]

Supplementary Material 5 Factor loadings of food groups for three dietary patterns by gender

Food group	Men (n=20,147)			Food group	Women (n=39,857)		
	Prudent pattern	Flour-based food and meat pattern	White rice pattern		Prudent pattern	Flour-based food and meat pattern	White rice pattern
Green and yellow vegetables	0.779	-	-	Green and yellow vegetables	0.789	-	-
Light-colored vegetables	0.733	-	-	Light-colored vegetables	0.710	-	-
Mushroom	0.620	-	-	Mushroom	0.616	-	-
Fish and shellfish	0.585	-	-	Fish and shellfish	0.587	-	-
Seaweeds	0.578	-	-	Seaweeds	0.585	-	-
Kimchi	0.532	-	-	Potatoes	0.480	-	-
Soybean pastes	0.438	-	-	Soybean pastes	0.472	-	-
Potatoes	0.424	-	-	Kimchi	0.462	-	-
Bean, tofu, and soymilk	0.343	-	-	Bean, tofu, and soymilk	0.460	-	-
Fruits	0.341	-	-	Fruits	0.385	-	-
Wheat flour and bread	-	0.596	-	Milk and dairy products	0.308	-	-
White meat and its products	-	0.525	-	Wheat flour and bread	-	0.632	-
Red meat and its products	-	0.522	-	Red meat and its products	-	0.597	-
Noodles and dumplings	-	0.482	-	White meat and its products	-	0.557	-
Eggs	-	0.416	-	Noodles and dumplings	-	0.521	-
Beverage	-	0.365	-	Sweets	-	0.385	-
Milk and dairy products	-	0.333	-	Eggs	-	0.346	-
Rice	-	-	0.947	Rice	-	-	0.930
Other grains	-	-	–0.930	Other grains	-	-	–0.914
Sweets	-	-	-	Beverage	-	-	-
Nuts	-	-	-	Nuts	-	-	-
Coffee and tea	-	-	-	Coffee and tea	-	-	-
Variance explained	17.02%	8.88%	6.64%	Variance explained	17.45%	8.34%	6.87%
Cumulative variance explained	17.02%	25.90%	32.55%	Cumulative variance explained	17.45%	25.79%	32.67%

*Factor loading absolute values ≥0.3 were included.
