# Supplementary material for: The association of dietary patterns with incident chronic kidney disease and kidney function decline among middle-aged Korean adults: a cohort study
Source: Epidemiol Health. 2023 Mar 21;45:e2023037. doi: 10.4178/epih.e2023037 (PMC10586924; doi:10.4178/epih.e2023037)
Supplement: Supplementary Material 6. — Food groups intakes (g/day) according to quartile of three dietary pattern scores found in Korean adults [file epih-45-e2023037-Supplementary-6.pdf]

Supplementary Material 6 Food groups intakes (g/day) according to quartile of three dietary pattern scores found in Korean adults

Food groups	Factor loading	Q1	Q2	Q3	Q4	*P* value
**Prudent pattern**						
Green and yellow vegetables	0.786	24.7 (14.8–37.9)	46.1 (32.3–62.9)	67.1 (48.3–90.3)	115.4 (80.2–166.5)	<0.0001
Light-colored vegetables	0.694	11.8 (6.5–18.5)	21.1 (14.1–31.0)	30.5 (20.4–44.1)	51.6 (32.4–81.8)	<0.0001
Mushroom	0.619	2.0 (1.0–3.5)	4.5 (2.0–7.4)	6.4 (3.2–12.9)	12.9 (6.4–30.0)	<0.0001
Seaweeds	0.595	0.6 (0.3–1.1)	1.2 (0.6–1.8)	1.5 (1.1–2.9)	3.1 (1.5–4.9)	<0.0001
Fish and shellfish	0.585	16.0 (9.4–24.8)	26.3 (17–38.8)	37.0 (24.3–54.2)	57.3 (36.2–87.2)	<0.0001
Kimchi	0.439	66.4 (31.2–125)	108.3 (62.3–166.3)	150.0 (81.9–198.0)	176.7 (110.2–270.0)	<0.0001
Potato	0.490	5.8 (2.5–10.9)	10.3 (5.5–18.2)	14.8 (8.6–26.3)	25.2 (11.7-–46.9)	<0.0001
Soybean paste	0.451	1.9 (0.8–1.9)	1.9 (1.9–4.5)	4.5 (1.9–4.5)	4.5 (2.3–7.1)	<0.0001
Bean, tofu, and soymilk	0.444	12.9 (5.0–19.3)	15.4 (12.9–30.4)	29.9 (13.3–46.0)	45.0 (20.6–73.1)	<0.0001
Fruits	0.409	82.6 (36.1–149.5)	142.4 (81.5–236.7)	204.9 (106.0–334.9)	280.6 (148.6–426.3)	<0.0001
Milk and dairy products	0.321	33.3 (10.7–103.0)	69.2 (18.7–166.0)	104.3 (30.0–204.3)	152.7 (48.9–254.4)	<0.0001
**Flour-based food and meat pattern**						
Red meat and its products	0.623	11.7 (5.0–20.8)	22.5 (13.3–36.7)	36.3 (21.7–56.7)	66.1 (38.6–104.4)	<0.0001
Wheat flour and bread	0.574	2.3 (0.0-–7.3)	7.5 (1.3–16.0)	14.2 (5.5–26.8)	30.4 (12.5–61.3)	<0.0001
White meat and its products	0.571	0.0 (0.0–2.5)	2.5 (0.0–2.5)	2.5 (1.3–6.3)	6.3 (2.5–9.4)	<0.0001
Noodles and dumplings	0.546	10.0 (0.0–25.7)	28.0 (10.0–45.8)	40.2 (23.7–73.5)	75.2 (37.3–130.8)	<0.0001
Sweets	0.394	0.4 (0.0–3.1)	3.5 (0.2–7.5)	5.3 (1.1–8.0)	7.5 (2.7–11.0)	<0.0001
Eggs	0.334	2.1 (0.0–10.7)	5.4 (1.7–10.7)	10.7 (4.2–16.1)	10.7 (5.4–25.0)	<0.0001
Beverage	0.316	0.0 (0.0–12.5)	5 (0.0–21.7)	12.5 (0.0–32.1)	21.7 (0.0–75.0)	<0.0001
**White rice pattern**						
Rice	0.938	5.0 (1.2–10.8)	3.7 (0.0–8.8)	5.0 (1.2–10.8)	660.0 (70.0–665.0)	<0.0001
Other grains	-0.922	660.0 (660.0–665.7)	660.0 (660.0–660.0)	440.0 (330.9–460.0)	0.0 (0.0–47.1)	<0.0001

Food groups were included when absolute values ≥0.3, and data on food intake were shown as 50% (25%–75%)
