# Supplementary material for: The association of dietary patterns with incident chronic kidney disease and kidney function decline among middle-aged Korean adults: a cohort study
Source: Epidemiol Health. 2023 Mar 21;45:e2023037. doi: 10.4178/epih.e2023037 (PMC10586924; doi:10.4178/epih.e2023037)
Supplement: Supplementary Material 7. — Hazard ratio of CKD incidence and kidney function decline incidence according to quartile of the white rice pattern score [file epih-45-e2023037-Supplementary-7.pdf]

Supplementary Material 7 Hazard ratio of CKD incidence and kidney function decline incidence according to quartile of the white rice pattern score

	Q1	Q2	Q3	Q4	*P* trend
Men (n)	5036	5037	5037	5037	
Person-years, sum	23992.4	24227.7	24293.6	24942.4	
**Risk of CKD**					
Cases, n	136	134	151	131	
Crude HR	1.00 (Ref)	0.97 (0.76, 1.23)	1.09 (0.86, 1.37)	0.87 (0.68, 1.10)	0.1486
Model 1	1.00 (Ref)	0.94 (0.74, 1.19)	0.97 (0.77, 1.23)	1.03 (0.81, 1.31)	0.5674
Model 2	1.00 (Ref)	0.89 (0.69, 1.14)	0.89 (0.69, 1.15)	0.97 (0.75, 1.26)	0.7072
Model 3	1.00 (Ref)	0.91 (0.71, 1.17)	0.91 (0.71, 1.18)	1.05 (0.81, 1.36)	0.3281
**Risk of kidney function decline ≥25%**					
Cases, n	100	116	110	100	
Crude HR	1.00 (Ref)	1.14 (0.87, 1.49)	1.07 (0.82, 1.41)	0.90 (0.68, 1.19)	0.1601
Model 1	1.00 (Ref)	1.11 (0.85, 1.45)	1.01 (0.77, 1.33)	0.99 (0.75, 1.31)	0.6967
Model 2	1.00 (Ref)	1.04 (0.78, 1.38)	0.91 (0.67, 1.22)	0.89 (0.66, 1.20)	0.3665
Model 3	1.00 (Ref)	1.07 (0.81, 1.42)	0.92 (0.68, 1.24)	1.04 (0.77, 1.40)	0.7915
Women (n)	9964	9964	9965	9964	
Person-years, sum	48246.7	48930.4	48539.8	50195.5	
**Risk of CKD**					
Cases, n	93	127	102	104	
Crude HR	1.00 (Ref)	1.32 (1.01, 1.72)	1.07 (0.8, 1.41)	1.00 (0.75, 1.32)	0.2667
Model 1	1.00 (Ref)	0.99 (0.76, 1.29)	1.02 (0.77, 1.36)	0.99 (0.75, 1.31)	0.9382
Model 2	1.00 (Ref)	0.92 (0.70, 1.21)	0.93 (0.68, 1.26)	0.88 (0.64, 1.21)	0.5595
Model 3	1.00 (Ref)	0.93 (0.70, 1.22)	0.97 (0.72, 1.33)	0.97 (0.71, 1.33)	0.9374
**Risk of kidney function decline ≥25%**					
Cases, n	115	146	125	159	
Crude HR	1.00 (Ref)	1.23 (0.96, 1.56)	1.05 (0.82, 1.35)	1.22 (0.96, 1.56)	0.2513
Model 1	1.00 (Ref)	1.05 (0.82, 1.34)	1.03 (0.80, 1.32)	1.21 (0.95, 1.54)	0.0819
Model 2	1.00 (Ref)	0.94 (0.73, 1.21)	0.89 (0.68, 1.17)	1.01 (0.77, 1.31)	0.5506
Model 3	1.00 (Ref)	0.95 (0.74, 1.22)	0.93 (0.71, 1.22)	1.07 (0.82, 1.40)	0.2743

Model 1: Adjusted for age.

Model 2: Further adjusted for body mass index, energy intake, education level, physical level (yes or no), drinking status, and smoking status.

Model 3: Further adjusted for diabetes, hypertension, dyslipidemia, proteinuria, and hyperuricemia.
