# Supplementary material for: The association of dietary patterns with incident chronic kidney disease and kidney function decline among middle-aged Korean adults: a cohort study
Source: Epidemiol Health. 2023 Mar 21;45:e2023037. doi: 10.4178/epih.e2023037 (PMC10586924; doi:10.4178/epih.e2023037)
Supplement: Supplementary Material 8. — Stratified analyses of associations between three dietary patterns and CKD risk and kidney function decline risk by gender [file epih-45-e2023037-Supplementary-8.pdf]

Supplementary Material 8 Stratified analyses of associations between three dietary patterns and CKD risk and kidney function decline risk by gender

Characteristic	Men				Characteristic	Women			
	HR-CKD	*P**	HR-Kidney function decline	*P**		HR-CKD	*P**	HR-Kidney function decline	*P**
Prudent pattern									
**Age (years)**		0.8501		0.9108	**Age (years)**		0.4466		0.4723
<55 (9,541)	1.27 (0.67,2.40)		0.80 (0.46,1.38)		<52 (18,032)	1.12 (0.45,2.78)		0.98 (0.63,1.52)	
≥55 (10,606)	0.91 (0.69,1.19)		0.63 (0.44,0.89)		≥52 (21,825)	0.82 (0.59,1.13)		0.80 (0.58,1.10)	
**BMI (kg/m^2)**		0.5646		0.5500	**BMI (kg/m^2)**		0.0541		0.0452
<25 (12,110)	0.91 (0.65,1.29)		0.61 (0.41,0.92)		<25 (28,802)	0.61 (0.40,0.94)		0.70 (0.51,0.98)	
≥25 (8,037)	0.79 (0.55,1.15)		0.63 (0.41,0.98)		≥25 (11,055)	1.24 (0.80,1.93)		1.14 (0.75,1.73)	
**Physical activity**		0.1446		0.2086	**Physical activity**		0.8057		0.7059
Inactive (15,331)	0.77 (0.57,1.05)		0.68 (0.48,0.96)		Inactive (31,404)	0.83 (0.58,1.18)		0.84 (0.63,1.13)	
Active (4,750)	1.34 (0.86,2.08)		0.80 (0.46,1.41)		Active (8,332)	1.19 (0.63,2.23)		0.91 (0.52,1.59)	
**Drinking status**		0.2546		0.7451	**Drinking status**		0.2462		0.1385
Non-drinker (5,552)	1.03 (0.67,1.58)		0.89 (0.54,1.49)		Non-drinker (27,933)	0.89 (0.63,1.25)		0.86 (0.64,1.15)	
Current drinker (14,532)	0.76 (0.56,1.03)		0.50 (0.34,0.72)		Current drinker (11,737)	0.89 (0.44,1.80)		0.83 (0.49,1.43)	
**Smoking status**		0.9378		0.0156					
Non-smoker (6,098)	1.02 (0.64,1.63)		0.62 (0.35,1.08)						
Ex-smoker (8,557)	0.71 (0.49,1.03)		0.59 (0.37,0.94)						
Current smoker (5,421)	0.86 (0.51,1.46)		0.70 (0.41,1.21)						
**Comorbidity**		0.4549		0.5337	**Comorbidity**		0.3131		0.4285
No (8,741)	0.88 (0.52,1.48)		0.54 (0.28,1.04)		No (23,422)	0.89 (0.50,1.56)		0.79 (0.53,1.19)	
Yes (11,095)	0.81 (0.61,1.08)		0.60 (0.43,0.84)		Yes (15,729)	0.87 (0.60,1.26)		0.88 (0.63,1.23)	
Flour-based food and meat pattern									
**Age (years)**		0.1462		0.2279	**Age (years)**		0.1192		0.3890
<55 (9,541)	1.07 (0.52,2.19)		1.03 (0.55,1.96)		<52 (18,032)	0.39 (0.15,1.03)		1.13 (0.68,1.88)	
≥55 (10,606)	2.01 (1.48,2.74)		1.61 (1.10,2.36)		≥52 (21,825)	1.43 (1.04,1.98)		1.58 (1.13,2.20)	
**BMI (kg/m^2)**		0.8471		0.5672	**BMI (kg/m^2)**		0.9159		0.5425
<25 (12,110)	1.69 (1.12,2.54)		1.31 (0.84,2.05)		<25 (28,802)	1.36 (0.84,2.21)		1.96 (1.37,2.80)	
≥25 (8,037)	1.34 (0.84,2.14)		1.78 (1.08,2.93)		≥25 (11,055)	1.70 (1.31,2.21)		1.35 (0.85,2.14)	
**Physical activity**		0.8945		0.2358	**Physical activity**		0.9746		0.3964
Inactive (15,331)	1.91 (1.34,2.72)		1.31 (0.88,1.95)		Inactive (31,404)	1.34 (0.91,1.97)		1.62 (1.17,2.23)	
Active (4,750)	1.56 (0.93,2.61)		2.11 (1.17,3.79)		Active (8,332)	1.42 (0.75,2.69)		1.95 (1.08,3.53)	
**Drinking status**		0.1953		0.1936	**Drinking status**		0.7251		0.9259
Non-drinker (5,552)	1.66 (1.01,2.74)		1.25 (0.69,2.26)		Non-drinker (27,933)	1.48 (1.03,2.13)		1.74 (1.27,2.40)	
Current drinker (14,532)	1.89 (1.32,2.71)		1.49 (0.99,2.24)		Current drinker (11,737)	0.73 (0.35,1.54)		1.00 (0.56,1.78)	
**Smoking status**		0.3651		0.0138					
Non-smoker (6,098)	2.44 (1.43,4.17)		1.75 (0.95,3.24)						
Ex-smoker (8,557)	1.63 (1.08,2.46)		1.68 (1.03,2.75)						
Current smoker (5,421)	1.59 (0.81,3.11)		1.07 (0.55,2.08)						
**Comorbidity**		0.6656		0.6395	**Comorbidity**		0.5391		0.3264
No (8,741)	2.05 (1.07,3.91)		1.25 (0.62,2.53)		No (23,422)	1.72 (0.89,3.33)		1.96 (1.24,3.08)	
Yes (11,095)	1.79 (1.28,2.50)		1.59 (1.09,2.33)		Yes (15,729)	1.20 (0.82,1.74)		1.45 (1.00,2.08)	
White rice pattern									
**Age (years)**		0.8555		0.9376	**Age (years)**		0.5762		0.0862
<55 (9,541)	1.21 (0.59,2.46)		1.02 (0.56,1.84)		<52 (18,032)	1.26 (0.52,3.08)		1.28 (0.81,2.02)	
≥55 (10,606)	1.00 (0.75,1.31)		0.98 (0.69,1.39)		≥52 (21,825)	0.79 (0.57,1.10)		0.85 (0.61,1.18)	
**BMI (kg/m^2)**		0.1529		0.2563	**BMI (kg/m^2)**		0.1848		0.2211
<25 (12,110)	0.76 (0.52,1.10)		0.79 (0.52,1.20)		<25 (28,802)	0.70 (0.45,1.10)		0.86 (0.61,1.21)	
≥25 (8,037)	1.20 (0.83,1.73)		0.97 (0.62,1.50)		≥25 (11,055)	1.09 (0.71,1.70)		1.31 (0.85,2.03)	
**Physical activity**		0.3541		0.1233	**Physical activity**		0.9097		0.4990
Inactive (15,331)	1.02 (0.75,1.40)		0.82 (0.58,1.16)		Inactive (31,404)	0.88 (0.62,1.25)		1.10 (0.82,1.48)	
Active (4,750)	0.82 (0.50,1.34)		1.16 (0.63,2.14)		Active (8,332)	0.89 (0.43,1.83)		0.67 (0.35,1.29)	
**Drinking status**		0.8257		0.6060	**Drinking status**		0.0018		0.1572
Non-drinker (5,552)	1.00 (0.63,1.57)		1.02 (0.59,1.74)		Nondrinker (27,933)	0.99 (0.69,1.41)		1.13 (0.83,1.55)	
Current drinker (14,532)	0.99 (0.71,1.37)		0.86 (0.60,1.24)		Current drinker (11,737)	0.72 (0.37,1.43)		0.81 (0.48,1.37)	

Characteristic	Men				Characteristic	Women			
	HR-CKD	*P**	HR-Kidney function decline	*P**		HR-CKD	*P**	HR-Kidney function decline	*P**
**Smoking status**		0.8213		0.4989					
Non-smoker (6,098)	0.81 (0.51,1.29)		0.51 (0.29,0.90)						
Ex-smoker (8,557)	1.05 (0.71,1.54)		1.15 (0.72,1.82)						
Current smoker (5,421)	0.91 (0.53,1.55)		0.75 (0.44,1.27)						
**Comorbidity**		0.8686		0.5320	**Comorbidity**		0.2769		0.5591
No (8,741)	0.79 (0.45,1.40)		0.79 (0.42,1.48)		No (23,422)	1.46 (0.78,2.74)		1.34 (0.88,2.06)	
Yes (11,095)	1.04 (0.77,1.40)		0.89 (0.63,1.26)		Yes (15,729)	0.74 (0.52,1.06)		0.79 (0.56,1.11)	

Comorbidity: diabetes, hypertension, dyslipidemia

Adjusted for age at baseline, body mass index, energy intake, education level, physical level (yes or no), drinking status, smoking status, and comorbidity; Variable will not be adjusted when this variable is stratified.
